# Exploring the Relationship Between Skeletal Muscle Mass and Muscle Strength in the Limbs of Elite Soccer Players

**DOI:** 10.3390/muscles5030050

**Published:** 2026-07-12

**Authors:** Valentina Cavedon, Chiara Milanese, Carlo Zancanaro

**Affiliations:** Laboratory of Anthropometry and Body Composition, Department of Neurosciences, Biomedicine and Movement Sciences, University of Verona, I-37131 Verona, Italy; valentina.cavedon@univr.it (V.C.); chiara.milanese@univr.it (C.M.)

**Keywords:** dual-energy X-ray absorptiometry, legs, arms, football, strength

## Abstract

Understanding the direct relationship between skeletal muscle mass and strength in athletes is paramount for optimizing performance. Such a relationship has been poorly investigated in soccer players. In this work, a large number (*n* = 225) of elite soccer players aged 14–37 years had whole-body (WB) and regional skeletal muscle mass (SMM) estimated from Dual-energy X-ray absorptiometry (DXA) scans, together with maximal isokinetic strength of several muscle groups. Results showed a statistically significant (*p* < 0.001) correlation between limb SMM or muscle strength and body mass (r = 0.84–0.86; r = 0.61–0.97, respectively) and stature (r = 0.58–0.64; r = 0.61–0.93, respectively), whereas the relationship with age was much more variable. A statistically significant (*p* < 0.001) correlation was found between SMM and muscle strength across all muscle groups (r = 0.55–0.80). Linear regression showed that SMM explained 30% to 64% of the variance in muscle strength. ANOVA showed that playing position had a statistically significant effect (*p* < 0.001) on all SMM and muscle strength values. Playing position had a significant effect on relative (normalized per-limb SMM) muscle strength during knee and ankle flexion. This work showed that DXA-measured skeletal muscle mass is associated with the strength of several limb muscle groups in soccer players. This association is of variable strength across muscle groups and is partially modulated by playing position.

## 1. Introduction

Muscle strength is an important factor of performance in most sports. The morpho-functional substrate for muscle strength is the neuromuscular system, of which muscle mass is a key factor. Skeletal muscle size and strength are positively related [1,2], so assessing the interaction between muscle mass and strength is paramount for trainers, coaches, and athletes to optimize an athlete’s power-to-weight ratio, track specific hypertrophic responses to training, and ensure safe recovery from injuries.

To properly evaluate the relationship between muscle mass and strength, direct measurement of muscle mass is essential, because scale weight and BMI fail to differentiate lean and fat tissue. Skeletal muscle mass (SMM) is better evaluated with computed tomography (CT) or magnetic resonance imaging (MRI) [3]. However, both techniques are costly and time-consuming, and computed tomography CT involves significant radiation exposure. Dual-energy X-ray absorptiometry (DXA) is a valid alternative to CT and MRI [3], being more widely available and less expensive than CT or MRI and involving very limited X-ray exposure. Further, DXA is more accurate than alternative methods such as bioelectrical impedance analysis or regression models [4] and has therefore emerged as a practical and widely adopted alternative to CT and MRI, providing valid estimates of regional and whole-body lean mass while maintaining high feasibility in sports settings.

Muscle strength is assessed with a variety of techniques [5]. Isokinetic dynamometers have been shown to provide valid and reliable measurements of muscle strength [6,7]. However, no consensus exists regarding the procedures to be applied, especially when both upper and lower limbs are to be investigated. Moreover, the limited availability of such facilities may hamper direct measurement of muscle strength, especially among younger athletes. Prediction equations are a practical tool to overcome this limitation. Work in [8] provided prediction equations to estimate maximal isokinetic muscle strength of major upper- and lower-limb muscle groups in healthy subjects, using easily measured variables such as age, body mass, and stature as independent variables.

In soccer, adequate SMM and limb strength are key factors in performance [9]; this is especially true for the lower limbs in outfield players, as they are instrumental for running, jumping, changing direction, and kicking [10]. Goalkeepers also rely on their upper arms for saving and throwing [11]. Muscle strength has been evaluated in large samples of soccer players [12,13]. However, most studies only involved one or two muscle groups and a limited number of participants. Similarly, lean mass has been explored in soccer players [14], with a few papers focusing on WB or segmental muscle mass [15,16,17]. However, very limited work has been done on the direct relationship between quantitative muscle variables and muscle strength in soccer players [18,19]. SMM is widely recognized as a determinant of muscle strength [1,2], although such a relationship may not be linear [20], particularly across different muscle groups. Clarifying this relationship in soccer players would improve the interpretation of DXA data and their application in athlete monitoring and performance assessment. In this work, we used DXA scanning and a skeletal muscle prediction equation [8] (see Materials and Methods section for details) to estimate SMM and muscle strength, respectively, in a large (n = 225) sample of elite soccer players aged 14–37 years with an aim at assessing the association of structural and functional muscle variables in several muscle groups and the effect thereupon of age, body mass, stature, and playing position. The following playing positions were considered: goalkeeper, GK; defender, D; midfielder, M; forward, F.

## 2. Results

### 2.1. Characteristics of the Study Sample

**Figure 1:**
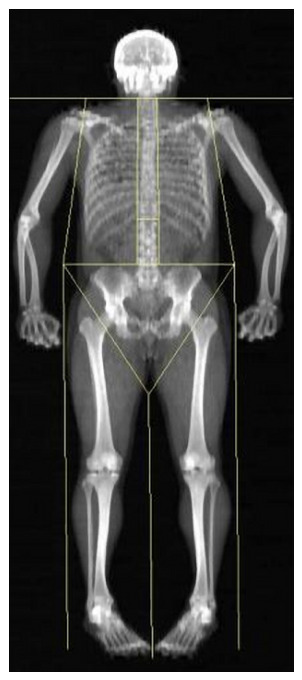
The typical segmentation of the DXA body image for regional body composition analysis. The main regions are the head, left and right arms, the trunk, the pelvis, and the left and right legs.

A total of 225 soccer players were included in the study. An example of the typical body segmentation used for regional body composition analysis is presented in Figure 1. In this work, the upper limbs variable was the sum of the left and right arm areas, and the lower limbs variable was the sum of the left and right leg areas. Please note that some muscular mass anatomically belonging to the arm or leg was excluded from the respective DXA area. The median age, body mass, stature, and BMI of the 225 participants were 18.0 y (IQ range: 2.0), 73.2 kg (IQ range: 11.35 kg), 179.9 cm (IQ range = 8.9 cm), and 22.6 km/m^2^ (IQ range: 2.07 kg/m^2^), respectively.

The mean values for skeletal muscle mass at the whole body, upper limbs, and lower limbs are reported in Table 1. In the entire sample, WB SMM was 42.2 ± 2.23% of body mass, upper limb SMM was 50.8 ± 1.5% of upper limb mass, and lower limb SMM was 60.1 ± 2.5% of lower limb mass.

### 2.2. Correlation Analysis

**Figure 2:**
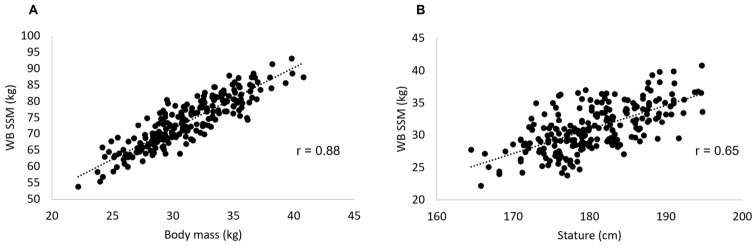
Scatterplot of the relationship between whole body (WB) skeletal muscle mass (SMM) and body mass (**A**) and stature (**B**). *p* < 0.001 for both. The dotted line represents the regression line.

WB SMM correlated at a very large strength with body mass (r = 0.88, R^2^ = 78%), at a large strengths with stature (r = 0.65, R^2^ = 42%) (Figure 2) and BMI (r = 0.65, R^2^ = 42%), and at a moderate strength with age (r = 0.40, R^2^ = 16%) (*p* < 0.001 for all). Similar results were obtained for upper limbs (r = 0.84, R^2^ = 71%; r = 0.58, R^2^ = 34%; r = 0.65, R^2^ = 42%; r = 0.45, R^2^ = 20%, respectively) and lower limbs (r = 0.86; R^2^ = 75%; r = 0.64, R^2^ = 42%; r = 0.62, R^2^ = 39%; r = 0.36, R^2^ = 13%, respectively) SMM (*p* < 0.001 for all). The correlation between SMM and playing position was r = 0.22 (*p* = 0.001), r = 0.25 (*p* < 0.001), and r = 0.20 (*p* = 0.002) for WB, upper limbs, and lower limbs SMM, respectively. R^2^ was 5%, 4%, and 6% (*p* = 0.001, *p* < 0.001, *p* = 0.002), respectively.

For all muscle groups, muscle strength correlated at moderate to almost perfect strength with body mass (r = 0.61–0.97, *p* < 0.001 for all; R^2^ = 38–94%) and stature (r = 0.61–0.93, *p* < 0.001 for all; R^2^ = 37–87%), and at small to moderate strength with BMI (r = 0.29–0.62, *p* < 0.001 for all; R^2^ = 0.1–39%). Age correlated with shoulder abduction (r = 0.24, *p* < 0.001; R^2^ = 5%), elbow flexion (r = 0.18, *p* = 0.006; R^2^ = 3%), wrist flexion (r = 0.24, *p* < 0.001; R^2^ = 6%), knee extension (r = −0.23, *p* < 0.001; R^2^ = 5%), ankle flexion (r = −0.43, *p* < 0.001; R^2^ = 18%) and, at the limit of statistical significance, knee flexion (r = 0.12, *p* = 0.075; R^2^ = 1%). No correlation was found between age and hip flexion (r = 0.01, *p* = ns) and hip extension (r = 0.01, *p* = ns).

**Figure 3:**
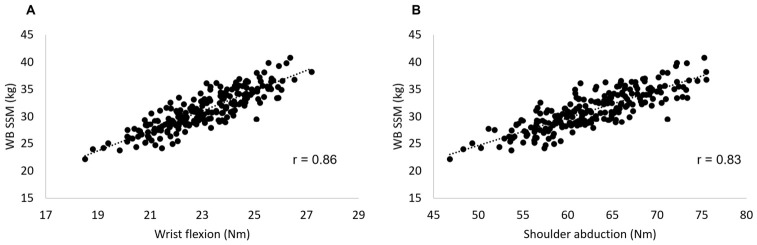
Scatterplot of the relationship between whole body (WB) skeletal muscle mass (SMM) and maximal isokinetic strength at wrist flexion (**A**) and shoulder abduction (**B**). *p* < 0.001 for both. The dotted line represents the regression line.

The bivariate correlation between DXA-derived SMM and maximal strength of several upper- and lower-limb muscle groups is reported in Table 2 (see a representative scatterplot in Figure 3), along with the results of linear regression of WB SMM on strength values. The strength of bivariate correlation was from large to very large.

### 2.3. Effect of Playing Position

Table 3 summarizes DXA-measured SMM and estimated maximal strength for several muscle groups in soccer players by playing position.

ANOVA conducted for age, body mass, stature, and BMI across playing positions showed a non-statistically significant difference for age and BMI (*p* > 0.05 for both; η^2^_p_ = 0.007 and 0.016, respectively) and a statistically significant difference for body mass and stature (*p* < 0.001 for both) with a large effect size (η^2^_p_ = 0.151 and 0.241), respectively. Post hoc analysis revealed that GKs were heavier and taller than all outfield positions (*p* < 0.001 for all), and Ds were heavier than Ms (*p* = 0.005) and taller than Ms and Fs (*p* < 0.001 for both).

**Figure 4:**
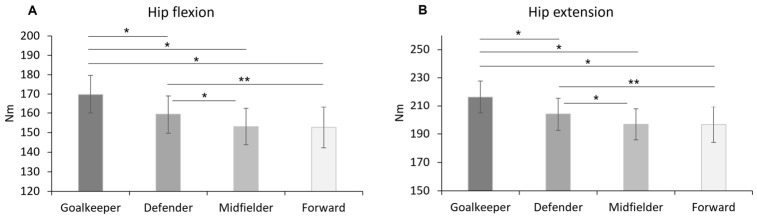
Maximal isokinetic strength at the hip for flexion (**A**) and extension (**B**) in the four playing positions. *, *p* < 0.05; **, *p* < 0.01.

ANOVA showed a statistically significant effect of playing position across all muscle mass and muscle strength variables (*p* < 0.001 for all), with medium to large effect sizes (η^2^_p_ = 0.084–0.227). The result of post hoc analysis is reported in Table 3 with a superscript; see also Figure 4.

GKs had higher SMM than Ds, Ms, and Fs at upper limbs (*p* = 0.004, *p* = 0.001, *p* = 0.001, respectively) as well as Ms at lower limbs (*p* = 0.005). At WB, GKs had higher SMM than Ms (*p* < 0.001) and Fs (*p* = 0.026). Ds had higher SMM than Ms at WB (*p* = 0.019), lower limbs (*p* = 0.022), and upper limbs (*p* = 0.036). GKs had greater muscle strength across all muscle groups than Ds, Ms, and Fs (*p* = 0.004–<0.001). Ds showed higher muscle strength than Ms for all muscle groups (*p* = 0.010–<0.001) except ankle flexion; Ds had higher muscle strength than Fs for all muscle groups (*p* = 0.006–<0.001) except wrist flexion, knee extension, and ankle flexion.

When age or BMI was introduced as a covariate, similar results were obtained. Introducing body mass in the model resulted in a statistically significant difference for lower limbs SMM (*p* = 0.009) across playing positions with a small effect size (η^2^_p_ = 0.051); muscle strength variables were statistically significantly different for all muscle groups (P from 0.031–<0.001) with a small-to-medium effect size (η^2^_p_ = 0.021–0.113) apart from ankle flexion (*p* = 0.192, η^2^_p_ = 0.021). When stature was used as a covariate, no statistically significant differences were found across playing positions for any skeletal muscle or strength variable. ANOVA conducted for relative muscle strength (normalized by WB SMM) across playing position showed a statistically significant effect for shoulder abduction (*p* = 0.025), knee flexion (*p* = 0.023), and ankle flexion (*p* = 0.011); the effect size was small for all (η^2^_p_ = 0.042−0.049). Post hoc analysis showed that GKs had higher muscle strength at shoulder abduction (*p* = 0.016) and knee flexion (*p* = 0.015) vs. Fs as well as lower muscle strength at ankle flexion vs. Ms. Relative muscle strength (normalized by upper or lower limbs SMM according to the specific muscles group) showed a significant effect of playing position for knee flexion (*p* = 0.012) with a small effect size (η^2^_p_ = 0.048) and ankle flexion (*p* = 0.027) with a small effect size (η^2^_p_ = 0.041). Post hoc analysis showed that GKs had greater knee flexor strength than Fs (*p* = 0.006).

## 3. Discussion

Results of this work represent the first assessment of the relationship between SMM and muscle strength in the limbs of soccer players. In a large (n = 225) sample of male elite soccer players aged 14–37 y, SMM and muscular strength of main upper- and lower-limb muscle groups were estimated using DXA-measured lean mass and MRI-validated predictive equations approximating the actual mass of skeletal muscle, and prediction equations, respectively. We found that this approach highlights several structure–function relationships in the limb musculature, which are partially modulated by demographic variables and playing position.

A first aim of this work was to investigate the relationships among SMM, muscle strength, and demographic variables in soccer players. DXA-derived SMM was associated with body mass, stature, and BMI at a very large to large strength at WB and in the upper and lower limbs, explaining 88–34% of variance. This was expected because SMM represents a major (42–60%) component of the entire body and the largest component of its appendages in the general population [21]. In turn, body mass was the primary independent variable in a regression analysis of the relationship between peak concentric moment of force at the knee and multiple anthropometric, demographic, and body composition variables in young male soccer players [22]. The relationship between SMM variables and age was much weaker, with an r value of moderate strength and a coefficient of determination ranging from 13% to 20%, despite the study sample including a large number of 20-year-old players. This is compatible with all participants being elite athletes with a high degree of muscularity, even in adolescence.

A second aim of this work was to explore the relationship between SMM and muscle strength in soccer players. We found that a strong relationship exists between upper- and lower-limb DXA-derived SMM and predicted isokinetic maximal strength of the upper and lower limbs, respectively. It should be underlined that the estimated maximal muscle strength values presented in this paper were consistent with those reported in previous studies of a young, athletic population [1,9,23,24,25] and generally higher than those shown in [8] in non-athletic males over a much larger age interval (15–83 y). In our sample, regional SMM explained 55–64% of upper-limb muscle strength variance and up to 61% of lower-limb variance. The proportion of explained variance in muscle strength explained by SMM could appear limited. However, evidence suggests that the relationship between muscle strength and muscle mass may not be linear [20].

The ability of SMM to explain muscular strength was rather homogeneous in the upper limb (R^2^ between 55% and 64% for all muscle groups) and much more variable in the lower limb (R^2^ ranging from 30% to 61%). In the lower limb, the lowest explained variance was for ankle flexion (30%), while similar values were found for hip flexion (R^2^ = 53%) and extension (R^2^ = 57%). When interpreting these data, it should be considered that the current positioning of the region of interest (ROI) in DXA images (Figure 1) excludes significant portions of the musculature involved in the shoulder and hip movement (e.g., the latissimus dorsi and the psoas major), thereby underestimating the actual muscle mass involved [26]. Moreover, some of the movements considered herein (e.g., wrist or ankle flexion) are carried out using a limited amount of the estimated upper or lower limb SMM, thereby overestimating the actual muscle mass involved. Further, obtaining maximal isokinetic strength could involve the contraction of accessory muscles outside the limb region. Consequently, muscle strength was correlated with WB SMM at the same or higher strength than with regional SMM (Table 2).

Interestingly, the correlation between muscle strength and age was statistically significant and negative for knee extension and ankle flexion. Longitudinal studies have shown increases in muscle strength with age among soccer players. The increase in muscle strength with age is well documented among children and adolescent soccer players [22,27,28,29]. However, when similar investigations were carried out in samples including adult players aged 33 and older [30], increasing age was associated with a limited or negative increase in extensor peak torque during concentric and eccentric contractions at the knee. Therefore, the presence of adult (>20-year-old; about 30% of the total) players in our sample could explain the above finding. The reason(s) for the negative correlation between strength and age only for knee extension and ankle flexion are not clear and deserve further investigation.

A third aim of this work was to assess the role of playing position in modulating the relationship between SMM and muscle strength. There is consistent evidence of positional differences in soccer players’ performance characteristics [31], physical fitness [32], body composition [14], and lower-limb strength [33]. In this work, we found that playing position affects both upper and lower limb SMM amount and muscle strength across all considered movements. In general, GKs (and, to a lesser extent, Ds) showed a larger amount of SMM as well as a higher maximal isokinetic peak force in the presence of similar ages and BMI. This was probably associated with larger body mass and stature in these playing positions. The above findings are consistent with previous data obtained in thigh muscles showing higher strength in GKs and Ds vs. Fs [33,34], and in contrast to those of [35], who found higher isokinetic strength in the hamstrings and quadriceps muscles of Ms vs. GKs. There is clearly a need for a more extensive, methodologically homogeneous investigation of muscle strength in soccer. Interestingly, a statistically significant difference in knee and ankle flexion across playing positions was observed for relative strength (adjusted for local muscle mass), indicating that these differences are independent of SMM size and may be playing-position specific.

This work has strengths and limitations to acknowledge. Strengths are the use of a robust sample of elite soccer players allowing the analysis of the relationship between strength and SMM per playing position, the accurate estimation of SMM from intramuscular fat-adjusted DXA data, and the simultaneous assessment of several muscle groups; limitations are the lack of direct strength measurement and the use of an equation developed in the general population to estimate strength in a sporting group, probably possessing a superior neuromuscular efficiency, thereby introducing a possible systematic bias. However, we were able to show in a small sample (n = 10) of players outside the studied sample that measured and predicted values of knee extension and flexion were not statistically significantly different (see Materials and Methods section below), thereby supporting the reliability of the finding presented therein. Future work using direct strength measurements in a larger sample of soccer players is required to confirm the findings presented herein.

## 4. Materials and Methods

### 4.1. Participants

A sample of male soccer players from the first team and the youth squads of an Italian Serie A club was extracted from our laboratory’s files. The age range was 14–37 years. Inclusion criteria were the absence of major injuries within the 6 months preceding measurement, the absence of acute illness, and no treatments that could influence body composition in the last year.

### 4.2. Procedures

All measurements were carried out in summer, before starting the conditioning period. The IRB approved the study at the University of Verona (2019-UNIVRCLR-0422326), and procedures conformed to the principles of the Helsinki Declaration.

WB DXA scans (QDR Horizon, Hologic, Marlborough, MA, USA; fan-beam technology, software version 13.6.05) were obtained according to the manufacturer’s specifications and those of [36]. The appendicular (=arms + legs regions, see Figure 1) lean soft tissue measured with DXA is a good proxy for skeletal muscle mass (SMM) [7] and has been calibrated against whole-body (WB) values assessed by CT or MRI [8]. Given the presence in the DXA-measured lean mass of unknown amounts of visceral tissue, water, and intramuscular fat, the actual SMM can be better approximated using the equation provided in [8] for the whole body (WB) and those provided by McCarthy et al. (2023) [37] for upper and lower limbs. All equations were validated against MRI.

Maximal strength (Nm) at the shoulder, elbow, and wrist, and at the hip, knee, and ankle was estimated using the equations proposed in [8] in 93 healthy males aged 15–83 years. The isokinetic peak torque for shoulder abduction, elbow flexion, wrist flexion, knee extension, knee flexion, ankle flexion, hip flexion, and hip extension was estimated from age, body mass, and stature. The relative muscle strength (N/m·kg^−1^) was obtained by dividing strength by WB or upper/lower limbs SMM as indicated. To test the validity of the equations proposed in [8], we measured in ten elite soccer players from the same Club who were not included in the investigated sample (mean age, 20.8 ± 4.5 y; mean body mass, 76.8 ± 5.5 kg; mean stature, 182.8 ± 4.3 cm) their knee extension and knee flexion strength using an isokinetic dynamometer (Cybex Norm, CYBEX International, Inc, Ronkonkoma, NY, USA) according to the protocol described in [8]. Measured and predicted values were compared by means of a paired samples t-test to check for systematic error. Measured and predicted muscle strength for knee extension were 233 ± 19.7 Nm and 222.1 ± 9.5 Nm, respectively. For knee flexion, measured and predicted values were 117.3 ± 32.0 Nm and 108.1 ± 7.2 Nm, respectively. The *t*-test showed a non-significant difference between measured and predicted values (knee extension, t = 2.073, *p* = 0.073; knee flexion, t = 1.287, *p* = 0.230).

### 4.3. Statistical Analysis

Bivariate correlation analysis was conducted, with Pearson’s correlation coefficient (r) calculated. The strength of correlation was rated according to [38] as follows: 0.1–0.29, small; 0.3–0.49, moderate; 0.50–0.69, large; 0.70–0.89, very large; 0.90–1.0, almost perfect. The coefficient of determination (R^2^) was calculated to estimate the percentage of variance in a dependent variable explained by an independent variable. For categorical variables (playing position), Spearman’s ρ was calculated. The effect of playing position on muscle strength was assessed with one-way ANOVA. The effect size was estimated using partial eta squared (η^2^_p_) and rated according to Cohen (1988) [39] as small (0.01), medium (0.06), or large (0.14). Post hoc analysis was carried out with Bonferroni correction. The Shapiro–Wilk test checked data normality. The IBM-SPSS statistical package (v. 26) was used for all analyses. Statistical significance was set at *p* ≤ 0.05.

## 5. Conclusions

This study is the first to investigate the relationship between skeletal muscle mass and strength across multiple upper- and lower-limb muscle groups in elite soccer players. Findings from 225 participants indicate that regional muscle mass is a major contributing factor to muscle strength. Its contribution is modulated by stature, muscle group, and playing position. Goalkeepers and defenders generally showed higher absolute values of both muscle mass and strength, supporting the presence of position-specific morphological and functional profiles.

DXA-derived regional body composition assessment may help practitioners to identify individual muscular profiles to be monitored for training-induced adaptations. In addition, the combined use of body composition data and strength prediction models may support position-specific conditioning strategies in elite soccer.

## Figures and Tables

**Table 1:** Whole body and limb skeletal muscle mass in 225 elite soccer players.

Site	Mean Skeletal Muscle Mass (±SD) (kg)	Minimum (kg)	Maximum (kg)	Median (kg)
Whole body	31.1 ± 3.5	22.2	40.8	30.8
Upper limbs	4.3 ± 0.5	2.9	5.9	4.3
Lower limbs	15.4 ± 1.8	11.1	20.2	15.2

**Table 2:** Isokinetic maximal strength in several muscle groups of upper and lower limbs and their bivariate (r) correlation with WB and limb skeletal muscle mass (SMM). The correlation of muscle strength with limb SMM is with upper limb SMM for shoulder abduction, elbow and wrist flexion, and lower limb SMM for knee extension and flexion, ankle flexion, and hip extension and flexion. The percentage of muscle strength variance explained by WB SSM (coefficient of determination, R^2^) is presented in parentheses. All correlations and coefficients of determination are statistically significant at *p* < 0.001.

Muscle Group	Maximal Strength (Nm)	Correlation with SMM r (R^2^)	
		WB	Limbs
Shoulder abduction	62.5 ± 5.7	0.83 (68%)	0.76 (58%)
Elbow flexion	54.4 ± 4.7	0.81 (66%)	0.74 (55%)
Wrist flexion	23.0 ± 1.6	0.86 (74%)	0.80 (64%)
Knee extension	219.6 ± 10.6	0.67 (44%)	0.63 (45%)
Knee flexion	104.4 ± 9.7	0.79 (62%)	0.78 (61%)
Ankle flexion	129.1 ± 4.1	0.54 (29%)	0.55 (30%)
Hip flexion	156.7 ±11.0	0.73 (53%)	0.73 (53%)
Hip extension	201.0 ± 12.9	0.75 (57%)	0.75 (57%)

**Table 3:** Mean ± SD value of skeletal muscle mass (SMM) and estimated maximal strength of several muscle groups in soccer players per playing position. The results of post hoc analysis across the four playing positions (Bonferroni correction) are indicated by superscript.

Variable	Goalkeeper (n = 22)	Defender (n = 73)	Midfielder (n = 87)	Forward (n = 43)
WB SMM (kg)	33.4 ± 3.0 ^+^°	31.7 ± 3.6 ^^^	30.1 ± 3.1	30.8 ± 3.7
Upper limbs SMM (kg)	4.8 ± 0.5 *	4.4 ± 0.5 ^^^	4.1 ± 0.4	4.2 ± 0.5
Lower limbs SMM (kg)	16.4 ± 1.5 ^+^	15.8 ± 1.9 ^^^	14.9 ± 1.6	15.3 ± 2.0
Shoulder abduction (Nm)	69.2 ± 4.6 *	63.9 ± 5.3 ^§^^	60.6 ± 4.7	60.7 ± 5.6
Elbow flexion (Nm)	60.0 ± 3.8 *	55.6 ± 4.3 ^§^^	52.8 ± 3.9	52.9 ± 4.6
Wrist flexion (Nm)	24.8 ± 1.3 *	23.3 ± 1.6 ^^^	22.5 ± 1.4	22.6 ± 1.7
Knee flexion (Nm)	115.9 ± 8.2 *	106.7 ± 8.8 ^^^	101.1 ± 8.1	101.1 ± 9.4
Knee extension (Nm)	231.0 ± 10.9 *	221.5 ± 9.6 ^§^	216.5 ± 9.1	216.8 ± 10.7
Ankle flexion (Nm)	132.8 ± 4.6 *	129.6 ± 3.8	128.1 ± 3.5	128.3 ± 4.2
Hip flexion (Nm)	169.8 ± 9.8 *	159.4 ± 9.7 ^§^^	153.1 ± 9.3	152.7 ± 10.5
Hip extension (Nm)	216.3 ± 11.5 *	204.1 ± 11.5 ^§^^	196.8 ± 10.9	196.7 ± 12.6

*, *p* < 0.05 vs. D, M, F; ^^^, *p* < 0.05 vs. M; ^+^, *p* < 0.01 vs. M; ^§^, *p* < 0.01 vs. F, °, *p* < 0.01 vs. F.

## Data Availability

The data presented in this study are available on request from the corresponding author.

## Abbreviations

The following abbreviations are used in this manuscript:

DXA	Dual-energy X-ray Absorptiometry
SMM	Skeletal Muscle Mass
CT	Computed Tomography
MRI	Magnetic Resonance Imaging
WB	Whole Body
GK	Goalkeeper
D	Defender
M	Midfielder
F	Forward
BMI	Body Mass Index
η^2^_p_	Partial eta squared
